# The development of Dutch COVID-19 ICU triage guidelines from an institutional work perspective

**DOI:** 10.1371/journal.pone.0291075

**Published:** 2023-09-14

**Authors:** Tamara Christina Broughton, Anne Marie Weggelaar-Jansen, Bert de Graaff

**Affiliations:** 1 Tranzo, Tilburg University, Tilburg, The Netherlands; 2 Erasmus School of Health Policy & Management, Erasmus University, Rotterdam, The Netherlands; The Open University, UNITED KINGDOM

## Abstract

**Introduction:**

Throughout the COVID-19 pandemic, two ICU triage guidelines were developed in the Netherlands—the Pandemic Guideline and the Guideline Code Black—ostensibly to tackle the threat of absolute care scarcity. Healthcare guidelines are generally based on evidence and prescribe what healthcare professionals should do in certain situations. We used the institutional work perspective, focusing on the human agency to create, maintain, and/or disrupt institutional structures, to study the development of these guidelines and observed that they did a lot more than just offering guidance to healthcare professionals. By including the Actor Network Theory (ANT) perspective on materiality’s agency in our theoretical lens, we show how guidelines, as a materiality—a non-human artefact—interact with human actors and as such shape and are shaped by the social context.

**Methods:**

17 online documents were analyzed. This analysis resulted in a timeline of events, which was used to identify key actors in the guideline development process. We included 12 purposely sampled respondents for semi-structured interviews. Interview transcripts were thematically coded.

**Results:**

During their development, the guidelines played a role in diverse forms of institutional work performed by a variety of stakeholders to: 1) strengthen the medical profession of intensivists; 2) control the medical profession; 3) gain support for the actions needed; and 4) protect the medical profession. In turn, institutional work performed by these stakeholders also shaped the guidelines, indicating the two-sidedness of the interaction between human actors and materiality in the healthcare context.

**Conclusions:**

This case study shows how guidelines as a materiality and human actors interact and influence each other in multiple ways, resulting in institutional work and thus shaping two institutions: the guidelines and healthcare professions. We found that a materiality does not stand on its own but influences and shapes institutional work in relation to human actors. By studying the development, implementation, and use of the guidelines, we gained more empirical insights into the impact materiality can have on the social context of healthcare and how this can influence existing institutional environments.

## Introduction

During the COVID-19 pandemic, many countries developed national triage guidelines (e.g. Pilbeam et al. [1]) as a reaction to the threat of insufficient intensive care (IC) capacity [2]. In the Netherlands, two ICU triage guidelines were developed: the Pandemic Guideline [3] and the Guideline Code Black [4, 5]. The Pandemic Guideline addresses the IC triage of patients in a crisis situation, based on medical criteria [3]. The Guideline Code Black describes the medical and ethical criteria for triage when there is still a shortage of IC beds after strict medical triage has been applied as described in the Pandemic Guideline (the code black phase) [4]. Generally, healthcare guidelines inform healthcare professionals of what they should do in certain circumstances based on scientific evidence [6], for example which treatment or diagnostic is suitable. However, the Dutch COVID-19 ICU guidelines are different as these describe a script for different phases of the crisis based on specific triage criteria, focusing on prioritizing health service delivery for all patients.

In this paper, we studied the development of the above-described guidelines, using institutional work as a theoretical lens, and explored how the guidelines influenced institutional work and therefore outranged content guidance. Institutional work is defined as the creation, maintenance, and disruption of institutional structures [7] and it outlines the agency of human actors to create or change institutions *and* social contexts. Thus, individual and groups of actors have an active role in institutional work [8, 9]. By using an institutional work perspective, we saw how the guidelines as materiality interacted with human actors and vice versa and thereby shaped the social context of healthcare. Although multiple definitions of materiality exist in literature, we define materiality in this study as a non-human artefact, object, or entity [10] which can have different forms such as technology (e.g. algorithm), methodology (e.g. framework), procedural (e.g. guideline), or physical (e.g. machine).

Previously, the role of materiality has rarely been discussed in this actor-centered approach to institutional theory in healthcare, despite previous work demonstrating its importance (e.g. Monteiro and Nicolini [11], Wallenburg et al. [10]). Materiality was described by Lawrence et al. [12] (p.1082) as “material artifacts…the common tools and techniques of strategizing and organizing and how are these used in practice… artifacts that instantiate established institutions to facilitate the transition between past habits and the elaboration of new habits for the future”. Studying the role of materiality in institutional work can show how a common tool, such as a healthcare guideline, shapes social context(s) and simultaneously is shaped by such contexts. As such, materiality plays a crucial part in important decision-making processes for institutional and societal change in healthcare. Scholars such as Lawrence et al. [12] and Jones et al. [13] have endeavored to introduce the dimension of materiality into our understanding of institutional work and institutional logics, but empirical evidence for this in healthcare is still lacking. This present study addresses this evidence gap.

To address this empirical gap and gain more insight into how materiality and human actors interact in institutional work in healthcare guideline development, we used the development of Dutch COVID-19 ICU triage guidelines as an in-depth case study. We followed the idea of Lawrence et al. [12] that materiality can play an important role in institutional work and applied this to the healthcare context. Our research question was: How does a materiality (in this case the guidelines) influence human actors and vice versa to shape the social context to create, maintain, and/or disrupt institutional structures in healthcare?

### Theoretical background

Institutional work originates from institutional theory and the sociology of practice [14]. Lawrence and Subbay [7] described institutional work as the practices of individual and collective actors aimed at creating, maintaining, and disrupting institutional structures. In institutional theory, institutional environments have been seen as relatively stable and homogeneous organizational fields that are shaped by institutional independent development rather than deliberate human agency [15–17]. In contrast to traditional institutional theory, institutional work states that human actors have the agency to create or change institutions from state A to state B. For example, Berghout et al. [14] showed that physicians do institutional work to reconfigure the medical profession, attributing clear agency to these professionals.

In literature there has been a clear call to clearly define what is meant by ’institution’ in context to the study [18, 19]. In this paper the definition of Scott [20] was used defining institution as ‘humanly devised rules, norms and beliefs that enable or constrain action and make social life predicable and meaningful’. In this case study, two institutions are at stake, the (development) of the COVID-19 triage guidelines and healthcare professions. Within the institutional theory paradigm, institutional work is one of the most actor-centric strands, and attributes influence or agency to human actors who can influence institutions and social contexts. However, the definition of human actors in institutional work neglects the role of materiality. Scholars such as Lawrence et al. [12] and Jones et al. [13] emphasized the importance of materiality in institutional processes and logics, and a few studies have since tried to incorporate materiality into institutional work (e.g. [21, 22]). Unfortunately, in these studies, material objects were described as accompanying actors in their performance of institutional work, with no agency of their own [23]. Taupin [23] did attribute agency to a non-human entity, the Atlantic Ocean, in institutional work, which changed the institution of surfing, and local planning in a coastal town offering a fresh way to consider materiality. Additionally, Pinch also expressed the importance of including materiality in institutional work in the following statement [24] (p.466): “Institutions have an inescapable material dimension part of the agency that materiality brings to institutions is the work of producing and reproducing (and sometimes changing) the social dimensions of institutions. Indeed, we neglect the material aspect of institutions at our peril, especially if we want to understand institutional change”. In this paper Pinch [24] for example refers to technology as an important component of materiality in institutional change. However, none of this work addresses the role of materiality in institutional work in healthcare.

To better understand the role of the guidelines in shaping institutional work and vice versa, we included the Actor Network Theory (ANT) perspective on materiality’s agency in our theoretical lens. It is especially relevant to add this since Latour [25] (p.71) defined actants as object of study, going beyond the notion of actors (as humans) as in institutional work. He refers to actants as: “anything that has an effect on another thing” and included both human and non-human entities in this definition. Additionally, Law [26] (p.156) defined actants as “all kinds of actors including objects, subjects, human beings, machines, animals, nature, ideas, organizations, inequalities, scale and sizes, and geographical arrangements”. In the ANT, agency is attributed not only to human actors but also to materiality (such as the guidelines in this case study). The ANT treats everything in the social and natural world as a web of relations that continuously affect each other. To better understand how materiality influences, these webs could be explored and characterized in individual case studies to understand the agency in discursively heterogeneous relations [26]. To better understand the influence of the guidelines as materiality in shaping the social healthcare context and vice versa, we researched the role and agency of the guidelines and how this interacted with others.

In healthcare guideline literature, research has focused on the development (e.g., [27–29]), implementation (e.g., [30]) and adherence to guidelines (e.g., [31, 32]). To our knowledge, an institutional work perspective has not previously been used to study (the development of) guidelines, and their impact on the social context of healthcare. Nevertheless, several authors showed that COVID-19 guidelines have an impact beyond giving advice on treatment and care. For example Bal et al. [33] have shown how guidelines are used as ‘mediating devices’ to add to the resilience of healthcare organizations during the pandemic. Additionally, Pilbeam et al. [34] illustrated how guidelines impacted the bodies, relationships and environments of healthcare workers throughout the pandemic in the UK. Our study contributes to this body of literature and supports the idea that guidelines have an influence on the social context of healthcare and vice versa.

## Materials and methods

To explore how the Dutch COVID-19 ICU triage guidelines were developed and how they affected institutional work, two researchers (TB, female and BdG, male) used qualitative research methods. Both researchers were trained in document study and conducting interviews by the university. Seventeen online documents published by medical associations and the Dutch government were analyzed to construct a timeline of events to identify key actors for interviews. This resulted in 12 purposely sampled semi-structured interviews, which were used to better understand how the guidelines interacted with human actors through institutional work during the development process.

For proper development and reporting of this study we complied to the ‘‘Consolidated criteria for reporting qualitative research” (COREQ) study design checklist and SRQR and COREQ reporting guidelines [35, 36].

### Data sources and data collection

Two authors (TB and BdG) searched independently for official national documents on the COVID-19 guidelines and their development on the internet, using the search terms: “triage guidelines”, “code black” and “COVID-19 guidelines”, followed by the snowballing method to find additional relevant documents. Documents were included if they were connected to the triage guideline development and were publicly available. Seventeen documents were included (see Table in S1 Table) and analyzed by open coding [37]. Based on the document analysis, a timeline of guideline development events (see Table in S2 Table) was drafted by one researcher (TB) and checked by another researcher (BdG). The aim of this timeline was to identify key actors and understand the processes of guideline development. The timeline ended after the publication of the most recent version of Pandemic Guideline (v. 2.1).

Based on the key actors identified, respondents were purposely selected. Respondents were identified as spokespeople, co-authors, or invited as a representation of an identified stakeholder. Selected respondents were invited by email that explained the study to participate and provided a written informed consent form. Almost all respondents agreed to participate or referred a colleague to participate (85%). One key stakeholder, the Dutch Federation of Medical Specialists, did not respond after multiple invitations, so was not included in this study. All respondents provided oral consent after the research methodology and data storage (compliant with the GDPR) were explained. The information and oral consent were repeated after consent and recorded and transcribed verbatim. This research was part of a broader research project called “Dancing with the virus”, which was ethically approved by the Research Ethics Review Committee of the Erasmus School of Health Policy & Management (20–08 Bal; 21–009 Bal). Data is available upon reasonable request considering the sensitivity of parts of our data, the understanding of the Dutch COVID-19 political context and especially to protect our respondents’ privacy and reputation.

Twelve semi-structured online interviews based on a predefined topic list derived from the theory and the timeline were conducted in April and June 2021 by one author (TB) (see Table 1 for the participants characteristics). After building a commencement relationship, topics discussed concerned the respondents’ involvement in guideline development, the (collective) work performed during this development, and the strategic use and purpose of the guideline development. The average length of the interviews conducted was 46 minutes.

**Table 1:** Participant characteristics.

Stakeholder group	Number of participants	Gender
Intensivists	1	Male
Triage committee member	2	Female / Male
Dutch government	1	Female
Medical ethicists	1	Male
Ethical advisory group	1	Female
Health and youth inspectorate	1	Female
Professional body of intensivists	3	Male
Overarching professional body of specialists	1	Male
ICU department manager	1	Female

### Data analysis

All interviews were video recorded and transcribed verbatim. Interview data were analyzed by thematic coding, as described by Braun and Clarke [38, 39], such that themes could be derived from the data obtained in an inductive way. After familiarization of the data, systematic coding was done by one researcher (TB) using ATLAS.ti (version 9) software. Initial themes were derived from the codes and were discussed within the research group to further develop and review the themes. Eventually, 17 themes were defined including themes such as “influence of guidelines”, “autonomy medical profession”, and “political interference”. Quotes were selected to illustrate the themes, which respondents checked and agreed to their use in this paper, which served as a member check.

## Results

While analyzing the data on the development of the Dutch COVID-19 ICU triage guidelines from an institutional work and ANT related materiality perspective, it became apparent that the guidelines and social healthcare context influenced each other. The guidelines were developed primarily to help hospitals prepare for a possible absolute scarcity in IC capacity due to a sudden influx of COVID-19 patients. As one respondent stated:

*“The objective of this [red*: *guideline] was to support the triage committees to prepare our hospitals for code black as much as possible*, *such that if code black occurs*, *we have a concrete implementation of the guidelines*, *so we can perform triage as well as we can*.”

*(Respondent 1)*.

The goals of the guidelines were: “*to aid IC care in times of crisis*” (Pandemic Guideline [3]) and “*to provide an ethical framework for difficult choices in case of absolute scarcity of IC capacity*” (Guideline Code Black [4]). However, the development of Guideline Code Black in particular was believed to be a “*theoretical exercise*” (respondent 3) and a “*discussion on paper*” (respondent 2), especially after the first wave of COVID-19 infections had passed.

This motivated us to research why the guidelines had been developed if absolute scarcity on a national level was not likely to happen. We discovered that the guidelines were used not only to prepare healthcare professionals and ultimately the country for a potential code black, but also are used in a more strategic way by different stakeholders.

### The role of the guidelines in strategically strengthening the medical profession

The professional body of intensive care physicians was in the lead to develop the Pandemic Guideline. Because of their central role in managing IC capacity in these guidelines and their already prominent role in the government infectious threat taskforce, the body was also closely involved in drafting the Guideline Code Black. They took on great responsibility during the pandemic and used this active role to strengthen their profession and their chances of becoming an independent medical profession. The professional body of intensive care physicians was established in 1997 in the Netherlands and does not have control over education or titles in their field. The chair of the body gained a lot of media attention from his role in developing the guidelines. He took part in talk shows on national television on an almost weekly basis, where he explained what was going on and what needed to be done. He was also prominent in the technical COVID-19 briefings given to the Dutch government. The chair used this prominent role to help the body become an independent medical profession:

*“There is more to the story; we as intensive care physicians are not an independent professional body yet*, *but we really want to become one*. *When I became chair of the professional body and we got our own quality standards*, *our goal was to become independent*. *Now we are still part of the internists and anesthetists’ professional body*, *and we are not a separate medical profession*. *[…] So*, *I wanted to show what we as intensivists could do*, *but I also did not want to step on anyone’s toes*.”

*(Respondent 11)*.

This quote, especially the reference to “*not stepping on anyone’s toes*”, shows how the chair tried to balance the act of displaying expertise and gaining more agency with not upsetting other medical professional groups. The chair was very tactical with his communication, as illustrated by the following quote:

*“I can remember when he [red*: *the chair of the professional body] was a guest at a talk show and he had a discussion with a politician [red*: *about the age criterium in the guideline] and admitted*: *ok we have to take another look at it [red*: *the age criterium in the guidelines]*. *In hindsight*, *he was very smart not to engage in an argument on television*. *At that moment*, *he stood no chance because he was politically too vulnerable*. *We [red*: *the members of the taskforce] weren’t happy about it*, *because we had all agreed upon the guideline after thorough consideration*. *[…] Eventually*, *the guideline was not changed at all*.”

*(Respondent 5)*.

### The role of the guidelines in controlling the medical profession

In the Netherlands, medical professionals commonly make decisions about the quality of care as part of their professional autonomy. Professional bodies develop guidelines, which describe the current scientific evidence on diagnosis and treatment. However, while the triage guidelines were being developed, non-medical criteria were also under discussion, especially the ethical criteria on age (based on the fair innings argument) in the Guideline Code Black. The age criterion in the guideline states that a younger person has a stronger claim to life-saving interventions than older patients do, thus prioritizing the younger generation over the older in situations of absolute scarcity [40]. This exposed the fair innings argument and opened it up for discussion by other stakeholders, especially politicians who did not agree with it at first. However, intensivists regard triage on age as part of their daily practice:

*“For us*, *the most important question was*, *if absolute scarcity occurs*, *which patient group would we deny access to the IC*? *And what hardly anyone*, *especially not the public*, *realized at that point*, *was that in normal situations we already select patients based on chance of survival and meaningful life after IC treatment and that age is a component of this [red*: *triage criteria]*. *And that [red*: *triage based on age] was an eye-opener for many*. *[…] Of course*, *we don’t admit people to the IC if they do not have a chance to survive*!”

*(Respondent 5)*

This standard intensivist practice was revealed in the triage guidelines and sparked a lot of debate about whether this is discrimination. Age was thus classified as an ethical rather than a medical criterion, which introduced actors from outside of the medical domain to the argument. The Dutch government immediately threatened to draft a law in which this criterion would be forbidden.

*“Discrimination based on age is unacceptable if you ask us [red*: *the government]*, *then we have to prepare a law to prohibit the discrimination*”

*(Respondent 3)*.

This shows how the guidelines triggered political action to control the decisions of the medical profession, even those decisions that were already a part of their daily professional practice. This clear observation of threat from the Dutch government to draft a law interfering with professional autonomy illustrates how the guidelines triggered other processes. This shows how different actors can influence each other by using their agency to advocate their opinion and influence the course of things. Interestingly, the law was never implemented because the government eventually agreed with age-based triage after the overarching medical association used the media (e.g., by appearing on television shows and giving interviews in newspapers) to gain more public support. They also gained societal support during the next discussed consultation rounds and during consultation with health regulators.

### The role of the guidelines in gaining support for the actions needed

As described in the previous section, age became an ethical issue in Guideline Code Black and gave the Dutch government a reason to interfere with the development of the triage guidelines. Discussions with different stakeholders on the importance of age in the triage guidelines convinced the Dutch government that societal support was needed. To achieve this, the Dutch government asked the overarching medical association for help. The government assumed that if all professional bodies represented by the medical association publicly agreed upon the criteria described in the two guidelines, then this would convince the Dutch population that the measurements were needed in code black situations. The overarching medical association agreed to assist but demanded consultation rounds with all stakeholders to gain the support needed. The Health and Youth Inspectorate, an intermediary governmental body in the Dutch healthcare system, formally asked the medical association to arrange and lead consultations with all relevant stakeholders [41].

*“The inspectorate believes that it is in the society’s interest to develop this guideline*. *However*, *it is only useful to make the guideline publicly available after some important stakeholders have taken note of the content*, *have discussed this*, *and have articulated to the authors how this guideline and the application of it will impact on their work and life*.”

*(Letter Health and Youth Inspectorate*, *2020-04-06)*.

The overarching medical body did not want to support the guidelines without these consultation rounds. The representative of the overarching medical association described these consultations as “*one of the pre-conditions to participate in the triage guidelines*” (respondent 9). In these consultation rounds, the guideline contents were discussed with many societal stakeholders, including elderly unions, patient associations, healthcare professional associations, and intermediary organizations. The above shows that the overarching medical association used these consultations rounds not only to gain support for the guidelines (as requested by the government) but also to enforce their own conditions. The association helped the government by gaining societal support for the two guidelines. This contributed to the acceptance of the guidelines and increased public support for government measures during the crisis.

### The role of the guidelines in protecting the medical profession

The guidelines also revealed how vulnerable the medical profession could be when faced with triage and deciding who will receive IC treatment and who will receive palliative care. The biggest fear here was legal liability.

*“There was also a legal side to the conversation because physicians were of course also very scared*, *because what if they make a [red*: *triage] decision*, *and there are complaints afterwards via criminal*, *disciplinary*, *or civil prosecution*, *because it is impossible to take away the right to file a complaint as a patient*. *Also*, *physicians have to feel safe enough to perform such a horrible act as triage [red*: *on non-medical grounds]*, *no one wants to do that*.”

*(Respondent 4)*.

This fear was described in multiple interviews by different medical professional stakeholders, and during the consultation rounds. To reduce this fear of prosecution, juridical protection was requested for medical professionals during triage situations. Consequently, The Health and Youth Inspectorate stated in a letter (dd. 2020-11-19) that *“the guidelines can be regarded as implementation of the standard of good care*, *as described by the Dutch Law”* [42]. This letter validated the guidelines and thus protected medical professionals from possible lawsuits after performing triage according to the guidelines. The branch organization of Dutch hospitals also expressed hospitals should not be held accountable for triage decisions (Respondent 5). In addition, the overarching medical association of physicians approached the Public Prosecutor’s Office to safeguard health professionals. The Public Prosecutor’s Office confirmed on 21 March 2021 that medical professionals following Guideline Code Black cannot be criminally prosecuted. The guideline was elevated as a standard of good quality care during times of exceptional crisis. This illustrates how the guidelines triggered the medical professional bodies to safeguard their liability and strengthen their legal position, and additionally how they used the guidelines to achieve this by gaining reactions of different important bodies.

### Institutional work shaping materiality

In the previous sections we showed how the guidelines as a materiality influenced work performed by a variety of stakeholders. However, our study also illustrated how institutional work, in turn, shaped the content and embedding of the guidelines. Both guidelines were described in one document at first, which was developed by the professional body of intensive care physicians. This document focused on medical triage criteria. However, the Dutch government got involved in the development of this guideline early on because they argued that code black was not solely a medical issue:

*“Politicians have seen this as a big question from the beginning*: *should only doctors be involved [red*: *in the guideline development] or should the society also think about it [red*: *who receives care during code black]*? *Because in that stage [red*: *code black] it’s a question of scarcity and thus not only a medical decision*. *Then code black is not a medical decision anymore*, *but more a social-cultural one*. *[…] However*, *it hasn’t been officially documented who is responsible for social-cultural issues in medical crisis situations*.”

*(Respondent 3)*.

Because the Dutch government became actively involved in the guideline development, which is normally part of the professional autonomy, the professional body of intensive care physicians felt pressure. To resolve this, the stakeholders eventually agreed that ethical criteria were outside the medical domain. One respondent described it as “a relief” (respondent 5) that an ethical advisory group took over drafting the ethical criteria of code black.

The ethical discussion about age was very difficult. Because of the different perspectives on the content of and responsibility for the guideline, both the professional body of intensive care physicians and the Dutch government called for a separation of the medical and non-medical criteria. They assumed that the ethical criteria would trigger a public debate, and both agreed that the Dutch government should take responsibility for the ethical criteria. These actions interacted and shaped the guidelines too.

## Discussion

Studying the development of the Dutch COVID-19 ICU triage guideline during the pandemic, using an institutional work and ANT materiality perspective, has shown how guidelines can do more than just giving guidance to healthcare professionals. We found that materiality (in this case the guidelines) can play an important role in shaping institutions in healthcare and therefore support and steer adjustments in institutional work structures. In institutional work, the agency of human actors influences the institutional context. In line with this, most research has focused on how institutional work occurs, who does institutional work, and what institutional work constitutes [12]. However, this does not address how materiality in relational interaction with human actors can influence institutional work.

This study provides empirical evidence how not only human actors but also materiality, such as guidelines, can play a role in shaping the social context of healthcare. In line with the work of Bal et al. [33] and Pilbeam et al. [34] we found that guidelines can play a role beyond giving guidance. For example, the professional body of intensivists used the guidelines strategically to strengthen their medical profession by drawing attention to their specific expertise while preventing liability actions for their normal triage procedures. The same guidelines triggered the Dutch government to gain control of age-based triage from the medical profession. They reframed these mundane triage processes as ethical instead of medical, placing it outside the profession and elevating it as something of high political relevance and in need of ethical consideration. These examples illustrate how the same materiality, the guidelines, influenced different kinds of interacting institutional work of different human actors while this shaped the social context of healthcare and the guideline itself.

Our findings are in agreement with those of other studies. For example, Wallenberg et al. [10] showed how rankings, as a materiality, can influence public service governance by inducing social actors to perform institutional work. The authors found that rankings create new expectations and requirements in healthcare organizations. However, this study did not focus on how the interaction between materiality and human actors creates agency, but more on how materiality has a one-sided influence. In another example, Raviola and Norbäck [22] showed how technology as a materiality triggers newsmakers in Italy to perform institutional work, emphasizing how action is taken during the interaction between humans and non-humans. Additionally, Lawrence et al. [12] included materiality in their study of institutional work and showed that human actors and materiality influence each other. They concluded that these relational interactions should be examined to fully understand how materiality and institutional work shape institutions. This paper has empirically contributed to the call by Lawrence et al. [12] and showed how institutional work is shaped by materiality in context of healthcare.

Our findings especially suggest that materiality should not be overlooked when studying institutional work in a complex institutional environment. We amplify the argument of Lawrence et al. [12], the work of Gawer and Phillips [21] and Raviola and Norbäck [22] and regard materiality as an important part of institutional work in the healthcare. All stakeholders in our case performed different forms of institutional work as part, or a result of the triage guidelines development. Each party involved safeguarded their own institutional background and in doing so, influenced societal opinion and processes. Including the guidelines as materiality in our study helped to show how these different forms of institutional work also interacted and influenced each other. For example, the political action to gain control over the medical profession by classifying the age criterion in the guidelines as an ethical, rather than a medical criterion, influenced the institutional work done by the overarching medical body to gain more support for the guidelines, since they realized the difficulty of the ethical discussion associated with it.

Whilst studying the development of the guidelines through an institutional work combined with ANT perspective and noticing the influence guidelines had on the healthcare context and vice versa, the broader definition of actants proposed by the actor network theory (ANT) was very useful. For example, our case study showed that a materiality (in this case the triage guidelines) revealed the juridical vulnerability medical professionals faced when performing triage. Medical professionals then used their agency to demand more protection, which resulted in the guidelines being regarded as the standard of good care. This broader definition of actants helped us to understand how materiality and human actors influence each other, shaping the healthcare context. This highlights that agency is not always a linear process (from state A to state B) as originally described in institutional work literature [7]. ANT helps us to understand that both human actors and materiality such as guidelines can be conceptualized as actants that can influence each other and shape social context(s) in a non-linear way. This can help to explain how guidelines trigger different and interacting forms of institutional work, depending on the human actor interacting with the guideline. In our case study for example, the guidelines triggered institutional work that protected medical professionals who needed to perform triage. Furthermore, the guidelines also triggered institutional work by the overarching professional body of physicians to gain more support for the actions needed to deal with the crisis. This illustrates the importance of how materiality is used by actors in different institutional contexts. The relational web of actants described in the ANT can explain these institutional work processes. The interaction between human actors and the materiality observed in this study raises the question of whether human actors and materiality should be regarded as separate or relational entities in institutional work in the future. There has been critique on combining institutional work and ANT in the past [43], because of clear differences in ontological foundations and epistemological commitments. However, this study does not aim to show how these two theories can be combined nor to evolve the existing theoretical bodies of institutional work and ANT. By being inspired by the broader definition of actants as commonly used in ANT and adding this to our theoretical lens, we included both human and non-human entities. Therefore, our empirical analysis of the development of the Dutch IC triage guidelines and what effect these guidelines have on the social context of healthcare was deepened.

By regarding the guidelines as actants and attribute them with agency throughout the development process, we observed that guidelines can serve as a boundary object as meant by Star and Griesemer [44] (p.409): an object that is part of multiple social worlds and facilitates communication between them. Boundary objects can thus help in the collaboration of different actors by bridging the differences between their social worlds or institutional background. In this case study, the guidelines played a role as boundary object, bringing all the key actors involved together and thereby boosting institutional work.

The broad definition of an object by Star and Griesemer [44] can also be useful in understanding our definition of materiality. In their work they use the term object for both science and pragmatist senses, as well as in the material sense as something: “Its materiality derives from action, not from a sense of prefabricated stuff or ‘‘thing”-ness. So, a theory may be a powerful object too” [45] (p. 603). This complements our definition of materiality and why we define guidelines as such.

More empirical research is needed in which materiality is included as an entity when trying to understand institutional work in a complex institutional environment such as in healthcare. Our case study has illustrated the profound effects a materiality can have and has shown that excluding materiality from empirical analysis hampers our understanding of how social contexts are (re)shaped. We focused on guideline development but found that this development triggered different forms of interacting institutional work depending on how different human actors viewed and used the guidelines, complicating linear views on such development.

There are three possible limitations of this study. First, this was a retrospective study in which key actors were interviewed after decisions about the COVID-19 triage guidelines had been made. This could have led to recall bias; for example, the key actors may not have accurately remembered the exact order of events or experiences they had during guideline development. Prospective and observational studies could improve our understanding of the interaction between a materiality and human actors during institutional work. Second, some important stakeholders (such as the Dutch Federation of Medical Specialists) were not interviewed during this study, so we may have missed additional examples of institutional work performed by these key actors triggered by the guidelines, which could have been an addition to the result section. Finally, by focusing on the agency of human actors and the developed guidelines as materiality, we have neglected many other non-human actors with agency during the pandemic. For example, the agency of the virus, as previously suggested by Latour [46] or the agency of the lockdown measures. To gain focus in this case study and dive into the agency of the guidelines as materiality, we have left these other agencies out the scope of this study. However, it would be very interesting to focus on different materiality’s that played a role in the COVID-19 pandemic and how these agencies interact.

## Conclusions

The aim of this study was to understand how guidelines as a materiality influenced human actors and vice versa to shape the social context as part of institutional work in healthcare. By studying the development of the Dutch COVID-19 ICU triage guidelines, we showed that the guidelines and human actors interacted and influenced each other in multiple ways, resulting in institutional work and thus shaping two institutions: the guidelines and healthcare professions.

First, the professional body of intensive care physicians used the guidelines strategically in their institutional work to strengthen their profession. Second, the guidelines triggered the Dutch government to take control of the ethical dimension of guideline development after age was described as a triage criterion in the guidelines. The Dutch government also used the guidelines as a materiality to gain societal support for the proposed measures with the help of the overarching medical association. Finally, the guidelines helped the medical professionals to shape their institutional environment by demanding more juridical protection in triage situations.

These findings show that the guidelines, as a materiality, did more than just describe what healthcare professionals should do in certain circumstances based on scientific evidence [6]. The guidelines influenced human actors and vice versa, and these interactions should be considered when developing new guidelines. The definition of actants proposed by the ANT (which attributes agency to ‘anything that has an effect on another thing’) and the concept of boundary objects (which play a role in bringing key actors together) has helped us understand that the guidelines -as a materiality- are also actants with agency that do not stand alone as a materiality, but rather interact with human actors to influence the social context of healthcare. Guideline developers and/or policy makers need to be aware of these interactions when developing new guidelines or changing existing guidelines, both of which happen regularly in healthcare. Taking these interactions into account will lead to a better understanding of how guidelines can influence existing institutional environments.

## Supporting information

S1 Table Overview of documents used document analysis. (DOCX)

S2 Table Timeline of decision-making process for the Dutch COVID-19 ICU triage guidelines. (DOCX)

S3 Table Abbreviations. (DOCX)
