# Supplementary material for: The development of Dutch COVID-19 ICU triage guidelines from an institutional work perspective
Source: PLoS One. 2023 Sep 14;18(9):e0291075. doi: 10.1371/journal.pone.0291075 (PMC10501561; doi:10.1371/journal.pone.0291075)
Supplement: S1 Table — (DOCX) [file pone.0291075.s001.docx]

# S*1* Table. Overview of documents used document analysis

| **Type of document** | **Author(s)* & source** |
| --- | --- |
| Triage guidelines | NVIC [1] |
|  | KNMG/FMS [2] |
|  | Local hospital** |
| Governance guideline | VWS & RIVM [3] |
| Letters | KNMG & FMS [4] |
|  | Chairman of NVIC [5] |
|  | Health and Youth Inspectorate [6,7] |
|  | Public Prosecutor’s Office [8] |
| Parliamentary letters | Minister of Health, Well-being and Sports [9] |
|  | Minister of Medical Care [10-14] |
| Political motions | Members of the House of Representatives [15] |
|  | Members of the House of Representatives [16] |

*See S3 table for definition of abbreviations

**The triage guideline of the local hospital was shared confidentially, so the source is not available.

**References:**

1. NVIC. Draaiboek pandemie deel 1 (v.2.1). 2021:1–24.

2. KNMG, FMS. Draaiboek ‘Triage op basis van niet-medische overwegingen voor IC-opname ten tijde van fase 3 in de COVID-19 pandemie.’. 2020:1–28.

3. Ministerie van Volksgezondheid, Welzijn en Sport en Landelijke Coördinatiestructuur Infectieziektebestrijding. Beleidsdraaiboek Influenzapandemie. 2004: 1-59.

4. Van Benthem PPG. Héman RACL. Draaiboek “Triage op basis van niet-medische overwegingen voor IC-opname ten tijde van fase 3 in de COVID-19 pandemie”. 2020 Nov 9. Available from: https://www.demedischspecialist.nl/sites/default/files/Aanbiedingsbrief%20Draaiboek%20Triage%20aan%20IGJ.pdf
5. Gommers D. Bericht van de voorzitter (1 t/m 17). 2020. Available from: https://nvic.nl/covid-19.
6. Eckenhausen MAW. Draaiboek Triage op basis van niet-medische overwegingen voor IC-opname ten tijden van fase 3 in de COVID-19 pandemie 2020.

7. Eckenhausen MAW. Kaljouw MJ. Aanbeveling ter voorbereiding op grotere druk op de zorg als gevolg van varianten Covid-19. 2021 Jan 26. Available from: https://www.rijksoverheid.nl/documenten/publicaties/2021/02/23/aanbevelingen-nza-en-igj-ter-voorbereiding-op-grotere-druk-op-de-zorg-als-gevolg-van-varianten-covid-19
8. Otte M. Aangiftes n.a.v. beslissingen in fase 3 C van het Draaiboek Triage o.b.v. van niet-medische overwegingen voor IC- in de COVID-19 pandemie. 2021 March 23. Available from: https://www.igj.nl/publicaties/brieven/2021/03/31/geen-strafvervolging-bij-overmacht-tijdens-%E2%80%98code-zwart%E2%80%99
9. de Jonge HM. Kamerbrief over stand van zaken coronavirus/ COVID-19. 2020 April 7. Available from: https://www.rijksoverheid.nl/documenten/kamerstukken/2020/04/07/kamerbrief-over-stand-van-zaken-covid-19

10. van Ark T. Antwoord op vragen van het lid Pia Dijkstra over de reactie van het kabinet op het “Draaiboek triage op basis van niet medische overwegingen voor IC-opname ten tijde van fase 3 in de COVID-19 pandemie.”. 2020.

11. van Ark T. Kamerbrief over status draaiboek “Triage op basis van niet-medische overwegingen voor IC-opname ten tijden van fase 3 in de COVID-19 pandemie”. 2020 Oct 2. Available from: https://www.rijksoverheid.nl/documenten/kamerstukken/2020/10/02/kamerbrief-over-status-draaiboek-triage-op-basis-van-niet-medische-overwegingen-ic-opname-fase-3-covid 19-pandemie

12. van Ark T. Kamerbrief over draaiboek “Triage op basis van niet-medische overwegingen voor IC-opname ten tijden van fase 3 in de COVID-19 pandemie”. 2020 Nov 24. Available from: https://www.rijksoverheid.nl/documenten/kamerstukken/2020/11/24/kamerbrief-over-draaiboek-triage-op-basis-van-niet-medische-overwegingen-voor-ic-opname-ten-tijde-van-fase-3-in-de-covid-19-pandemie
13. van Ark T. Draaiboek “Triage op basis van niet-medische overwegingen voor IC-opname ten tijden van fase 3 in de COVID-19 pandemie”. 2021 Jan 4. Available from: https://www.rijksoverheid.nl/documenten/kamerstukken/2021/01/04/kamerbrief-over-draaiboek-triage-op-basis-van-niet-medische-overwegingen-voor-ic-opname-ten-tijde-van-fase-3-in-de-covid-19-pandemie
14. van Ark T. Kabinetsstandpunt Triage op basis van niet-medische overwegingen voor IC-opname ten tijden van fase 3 in de COVID-19 pandemie”. 2021 Jan 11. Available from: https://www.rijksoverheid.nl/documenten/kamerstukken/2021/01/11/kamerbrief-over-kabinetsstandpunt-draaiboek-triage-op-basis-van-niet-medische-overwegingen-voor-ic-opname-ten-tijde-van-fase-3-in-de-covid-19-pandemie
15. Krol HCM. Hijink, M. Motie van leden Krol en Hijink over geen leeftijdsgrenzen hanteren voor intensieve careafdelingen [Political motion nr. 25295-142]. 2020 March 12. Available from: https://www.tweedekamer.nl/kamerstukken/detail?id=2020Z05034&did=2020D10427
16. Jetten R et al. Motie van lid Jetten c.s. over het draaiboek “triage op basis van niet niet-medische overwegingen voor IC-opname ten tijde van fase 3 in de COVID-19 pandemie" [Political motion nr. 25295-860]. 2021 Jan 5. Available from: https://www.tweedekamer.nl/kamerstukken/moties/detail?id=2021Z00110&did=2021D00537
