# Supplementary material for: The development of Dutch COVID-19 ICU triage guidelines from an institutional work perspective
Source: PLoS One. 2023 Sep 14;18(9):e0291075. doi: 10.1371/journal.pone.0291075 (PMC10501561; doi:10.1371/journal.pone.0291075)
Supplement: S2 Table — (DOCX) [file pone.0291075.s002.docx]

# S2 table*.* Timeline of decision-making process for the Dutch COVID-19 ICU triage guidelines

| **Date** | **Milestone** | **Actors involved*** |
| --- | --- | --- |
| ***2004-07*** | *Publication governance document “Influenza pandemic”* | Minister VWS (Bruins) |
| ***2014-08-21*** | *Publication Triage Consensus Statement in CHEST* | Christian et al. |
| ***2018-09-24*** | *Publication Pandemic Guideline v. 1.0* (not COVID-specific) | NVIC |
| ***2019-05*** | *Meeting of infectious threat taskforce to discuss the drafting of Pandemic Guideline* | Infectious threat taskforce of NVIC |
| **2019-12** | WHO picks up a media statement by the Wuhan Municipal Health Commission from their website on cases of “viral pneumonia” | WHO |
| **2020-02-27** | First officially reported case of COVID-19 in the Netherlands | Minister VWS (Bruins) informs House of Representatives |
| **2020-03-06** | Publication Pandemic Guideline v. 1.2 (first COVID-specific version of the guideline) | NVIC |
| **2020-03-11** | COVID-19 officially declared a pandemic | WHO |
| **2020-03-12** | Motion Krol and Hijink, no age discrimination on IC admission accepted unanimously | House of Representatives |
| **2020-03-16** | Publication Pandemic Guideline v. 1.3 | NVIC |
| **2020-03-19** | Minister Bruins steps back as Minister of Health Welfare and Sport, Minister Hugo de Jonge is installed | VWS |
| **2020-03-20** | Signal from Hospital Bernhoven – IC-capacity is very scarce in local hospital | Hospital to KNMG and FMS |
| **2020-04-03** | Publication Pandemic Guideline v. 1.4 | NVIC |
| **2020-04-07** | Letter from government to House of Representatives about the age-criteria in Guideline Phase 3C | Minister VWS (de Jonge) |
| **2020-04-07 –2020-04-14** | Consultation of KNMG with directors of important professional groups | KNMG, ActiZ, ANBO, KBO-PCOB, LHV, NFU, NHG, NOOM, NVAVG, NVZ, Patient Federation Nederland, V&VN, Verenso, VGN |
| **2020-05-22** | Publication Pandemic Guideline v. 2.0 | NVIC |
| **2020-06-16** | Publication Guideline Phase 3C v. 1.0 | FMS, KNMG |
| **2020-06-16** | Letter to House of representatives by Minister van Rijn as reaction to Guideline Phase 3C | Minister of Medical Care (van Rijn) |
| **2020-Summer** | Consultation with stakeholders about Guideline Phase 3C | FMS, KNMG. NVIC, ANBO, KBO-PCOB, KBO Brabant, NOOM |
| **2020-09-22** | RIVM announces second wave of COVID-19 | RIVM |
| **2020-11-02** | Publication Guideline Phase 3C v. 2.0 | FMS, KNMG |
| **2020-11-19** | Letter from Health and Youth Inspectorate directed to FMS and KNMG confirming Guideline Phase 3C is regarded as “implementation of the standards of good care as described by the Dutch Law” | Inspector general (Eckenhausen) |
| **2021-01-04** | Letter to House of Representatives about the fair innings argument (the age-based criterion for triage) | Minister of Medical Care (van Ark) |
| **2021-01-05** | Guidelines addressed in plenary meeting  Motion Jette: reconsider point of view of the government about Guideline Phase 3C | House of Representatives |
| **2021-01-11** | Letter to House of Representatives confirming the political support of Guideline Phase 3C | Minister of Medical Care (van Ark) |
| **2021-02-15** | VWS asks Dutch hospitals to prepare for a possible code black via the ROAZ | VWS, ROAZ, Dutch hospitals |
| **2021-02-23** | Technical briefing about Guideline Phase 3C | FMS, KNMG, NVIC |
| **2021-03-19** | Publication Pandemic Guideline v. 2.1 | NVIC |
| **2021-03-21** | Letter from Public Prosecution Office confirming that medical professionals cannot be criminally prosecuted when following Guideline Phase 3C | Public Prosecution Office (Otte) |

*See Table in S3 Table for definitions of abbreviations
