# Supplementary material for: The development of Dutch COVID-19 ICU triage guidelines from an institutional work perspective
Source: PLoS One. 2023 Sep 14;18(9):e0291075. doi: 10.1371/journal.pone.0291075 (PMC10501561; doi:10.1371/journal.pone.0291075)
Supplement: S3 Table — (DOCX) [file pone.0291075.s003.docx]

# S3 Table*.* Abbreviations

| **Abbreviation** |  |
| --- | --- |
| ActiZ | Branch organisation for nursing care, residential are and home care |
| ANBO | Dutch General Union for Elderly |
| FMS | Federation of Medical Specialists |
| IGJ | Health and Youth Inspectorate |
| KBO | Catholic Union for Elderly |
| KNMG | The Royal Dutch Society for the Promotion of Medicine |
| LHV | National Association of General Practitioners |
| NFU | Dutch Federation of Academic Medical Centres |
| NHG | Dutch Society of General Practitioners |
| NOOM | Network of Organisations of Older Migrants |
| NVAVG | Dutch Association of Physicians for the Mentally Disabled |
| NVIC | Dutch Association for Intensivists |
| NVZ | Dutch Association of Hospitals |
| PCOB | Protestant Christian Union for the Elderly |
| RIVM | National Institute for Public Health and Environment |
| ROAZ | Regional Coalition of Intensive Care |
| V&VN | Dutch Nurses and Caretakers |
| VGN | Dutch Association of Care for the Disabled |
| VWS | Ministry of Health, Welfare and Sports |
| WHO | World Health Organisation |
